# Noncommunicable chronic diseases and health challenges in 2050

**DOI:** 10.1590/1980-549720260011

**Published:** 2026-03-16

**Authors:** Deborah Carvalho Malta, Guilherme Augusto Veloso, Crizian Saar Gomes, Maurício Lima Barreto

**Affiliations:** I Universidade Federal de Minas Gerais, Nursing School, Department of Maternal-Child Nursing and Public Health - Belo Horizonte (MG), Brazil.; II Universidade Federal Fluminense - Niterói (RJ), Brazil.; III Universidade Federal de Minas Gerais, School of Medicine, Graduate Program in Public Health - Belo Horizonte (MG), Brazil.; IV Fundação Oswaldo Cruz, Center for Data and Knowledge Integration for Health, Instituto Gonçalo Moniz - Salvador (BA), Brazil.

**Keywords:** Chronic noncommunicable diseases, Risk factors, Global burden of disease, Brazil

## Abstract

**Objectives:**

The study aims to analyze the burden of diseases related to noncommunicable chronic diseases (NCDs) and their risk factors (RFs) from 1990 to 2021 and to project of NCDs and RFs for the years 2030 and 2050.

**Methods:**

Estimates of risk factors and mortality from NCDs in Brazil and its States were analyzed based on data from the Global Burden of Disease Study.

**Results:**

There was a 37.7% reduction in premature mortality rates from NCDs and a 34% reduction in Disability-Adjusted Life Years (DALYs) between 1990 and 2021. Projections for 2050 indicate that mortality from cardiovascular diseases will continue to decline and that these will be supplanted by neoplasms. Mortality rates from diabetes tend to increase, while chronic respiratory diseases show a downward trend. In 2021, the main risk factors associated with premature deaths from NCDs were: high blood pressure, smoking, poor diet, high Body Mass Index (BMI), and high fasting glucose. Projections through 2050 point to an increase in risk factors such as obesity, high blood pressure, high glucose, physical inactivity, and poor diet - which may compromise the achievement of the 2050 targets for reducing premature mortality from NCDs.

**Conclusion:**

The study points out that global targets may not be achieved, raising an alert to the urgent need to strengthen public policies aimed at the prevention and control of NCDs.

## INTRODUCTION

Noncommunicable chronic diseases (NCDs) are the leading cause of global morbidity and mortality, resulting in premature deaths, disabilities, loss of quality of life, economic impacts, and burden on health systems^1^ ^,^ ^2^. NCDs are estimated to account for 75% of global mortality^3^ .

The four main groups of NCDs - cardiovascular diseases (CVD), cancer, chronic respiratory diseases, and diabetes - share common and modifiable behavioral risk factors (RF), such as smoking, alcohol abuse, physical inactivity, and poor diet^4^ ^,^ ^5^. In addition to these, metabolic factors such as high blood pressure, obesity, high cholesterol, and high fasting plasm glucose also stand out^2^ ^,^ ^5^ ^,^ ^6^. Socioeconomic determinants play a strong role in the causality network of NCDs and their RF^1^ ^,^ ^7^ ^,^ ^8^.

The 2030 Agenda for Sustainable Development established a goal of reducing the “unconditional probability of premature death from NCDs” by one-third^9^. To date, only 19 countries - representing only 2.7% of the world’s population - are on track to achieve this goal. At the current pace, it would take approximately 45 years to achieve this goal^10^.

In Brazil, studies indicate that mortality rates from NCDs showed a downward trend between 2010 and 2015. However, this progress was interrupted in 2016 and worsened during the COVID-19 pandemic, compromising the possibility of achieving the 30% reduction target set by *Plano de DANT*, a Brazilian strategic action plan to tackle CNCDs^2^ ^,^ ^11^ ^,^ ^12^ ^,^ ^13^ .

In the face of persistent global challenges, new commitments have been proposed. In 2024, the *Lancet Commission* launched the goal of reducing the probability of premature death from selected diseases by 50% by 2050 - a proposal that became known as “50 by 50.” This goal can be achieved by addressing 15 priority conditions - 8 related to infectious diseases and maternal health, and 7 related to NCDs and injuries^14^.

The main proposals for achieving the 2050 targets would be

a) concentrating resources on priority conditions, including NCDs, with a view to maximizing the impact on reducing premature mortality;
b) increasing global health funding;
c) tobacco control as a priority strategy, with tax increases, marketing bans, and other measures;
d) reducing poverty and social inequalities;
e) improving access to primary care and essential medicines;
f) promoting healthy eating, physical activity, and healthy environments.

In this context, the *Brazil Saúde Amanhã* initiative, launched by Fundação Oswaldo Cruz (Fiocruz), stands out. It is based on the guiding question: *“What is the future of health in Brazil?*”. The initiative coordinates a multidisciplinary research network focused on investigation, strategic prospecting, and the formulation of public policies for the health sector, strengthening the Unified Health System (SUS)^15^ ^,^ ^16^.

This study falls within this scope and has the following objectives: to analyze the burden of diseases related to NCDs and their RF in the period from 1990 to 2021; and to project the evolution of NCDs and RF for the years 2030 and 2050.

## METHODS

This study analyzed estimates of RF and mortality from NCDs in Brazil and its States, based on data from the 2021 Global Burden of Disease (GBD) Study, from the Institute for Health Metrics and Evaluation (IHME), available at: http://ghdx.healthdata.org/.

### Mortality

The GBD mortality estimates are based on data from the Mortality Information System (SIM) of the Ministry of Health^17^, with adjustments for so-called *garbage codes*, using algorithms for redistributing the underlying causes of death, considering age, sex, and year^18^ ^,^ ^19^.

The unconditional probabilities of premature death from NCDs were calculated. For each age group (𝑥 𝑥 +5), the probability of death during the interval was estimated based on the age-specific mortality rate, denoted by *m*_*x*_ , using the approximation $q_{x}=\frac{{5m}_{x}}{1+{2,5m}_{x}}$. The unconditional probability of death was estimated as:

$$P\left(30\leq Age\;of\;death\leq 69\right)=1-\prod_{x=30}^{65}\left(1-p_{x}\right)$$

The metrics used to analyze the burden of NCDs in Brazil was: absolute number of deaths, age-standardized mortality rates, and years of life lost due to premature death and Disability-Adjusted Life Years (DALYs). These metrics were presented for all age groups and for premature deaths between 30 and 69 years of age, according to the World Health Organization (WHO) Global Plan^1^. The analyses covered all diseases and the four main groups of NCDs in Brazil in 1990 and 2021, as well as the percentage change (PC) over the period.

To verify subnational inequalities, premature mortality rates from NCDs were analyzed for the 26 Brazilian States and the Federal District in 1990 and 2021.

### Risk factors

The main sources of data on RF for the Brazilian population used by the GBD were the National Health Survey (PNS), the Surveillance System for Risk and Protective Factors for Chronic Diseases (Vigitel), and the National School Health Survey (PeNSE)^13^ ^,^ ^20^.

The GBD study uses a hierarchical list of risk factors organized into four levels, ranging from broad groups (metabolic, behavioral, and environmental) to more detailed ones, totaling 87 factors analyzed in 2021^21^.

Regarding RF, the Summary Exposure Value (SEV) was analyzed. The SEV represents the prevalence of exposure weighted by relative risk, ranging from 0% to 100%, in which 0% indicates a total absence of exposure and 100% corresponds to the maximum level of exposure observed in the population. A reduction in SEV indicates a decrease in population exposure to the risk factor, whereas an increase suggests the opposite. More information on SEV is available in other publications^20^ ^,^ ^22^.

### Projections for 2050

#### Risk factors

SEV trends for risk factors related to NCDs between 1990 and 2021 were analyzed, including: smoking, poor diet, low physical activity, excessive alcohol consumption, high blood pressure, high cholesterol, impaired kidney function, high fasting blood glucose, high Body Mass Index (BMI), air pollution, and temperature. Based on the evolution observed during this period, projections for these indicators were calculated up to the year 2050.

#### Mortality projections

Projections were calculated for premature mortality rates and mortality rates at all ages by NCDs and their four main groups, as well as the probability of premature death for the years 2030 and 2050. These results were compared to the Sustainable Development Goals (SDGs) targets agreed upon in 2015, which estimate a 30% reduction in the unconditional probability of premature death from NCDs^23^. Projections were also made to compare with the target proposed by the Lancet Commission (50% reduction by 2050)^14^. The values observed from the years 2015 to 2021 were used as a reference for the projection calculations.

An ETS model was used. The term refers to the class of models that combine three fundamental components: Error (E), Trend (T), and Seasonality (S); of the type: Additive (A), Additive Trend (A), and No Seasonality (*N*) (AAN). This model is especially suitable for time series that exhibit a trend but do not show seasonal patterns. In addition, a dampened trend was used, which introduces a dampening factor to gradually reduce the influence of the trend on long-term projections.

The analyses were conducted in RStudio (RStudio Team, 2019) and the figures were produced using the ggplot214 package.

#### Ethical considerations

The GBD-Brazil project was approved by the Research Ethics Committee of Universidade Federal de Minas Gerais (UFMG), registered under Project number CAAE - 62803316.7.0000.5149.

## Data availability statement

All data supporting the results of this study are available at: http://ghdx.healthdata.org

## RESULTS

### Mortality

Table 1 shows the mortality and DALY burden caused by NCDs in Brazil in 1990 and 2021. Premature mortality rates from all causes decreased by 7.1%. In the same period, there was a 37.7% reduction in premature mortality rates from NCDs and a 34% reduction in DALY rates. Between 1990 and 2021, premature mortality rates from CVD, chronic respiratory diseases, diabetes mellitus, and neoplasms decreased by 52.7%, 38.5%, 24%, and 12.5%, respectively. DALY rates showed similar reductions (Table 1).

**Table 1.** Number of deaths and standardized mortality and DALY rates in the population aged 30-69 years and in all ages, for both sexes, in Brazil, in 1990 and 2021.

Cause of death	Age group	Number of deaths			Standardized mortality rate (per 100 thousand)			Standardized DALYs rate (per 100 thousand)		
		1990	2021	PC	1990	2021	PC	1990	2021	PC
Todas as causas	30-69 years	350,153	804,109	129.6	792.1	735.8	-7.1	44,647	42,524	-4.8
	All ages	875,709	1,796,921	105.2	926.4	742.9	-19.8	43,050	33,056	-23.2
NCD	30-69 years	212,908	342,930	61.1	502.6	312.9	-37.7	18,103	11,942	-34
	All ages	433,065	788,379	82	542.2	321.8	-40.7	13,261	8,500	-35.9
Diabetes mellitus	30-69 years	13,191	26,289	99.3	31.5	24	-24	1,656	1,627	-1.7
	All ages	26,319	64,839	146.4	32.1	26.5	-17.5	1,104	1,080	-2.2
Cardiovascular diseases	30-69 years	121,486	148,709	22.4	286.7	135.7	-52.7	9,962	4,902	-50.8
	All ages	258,237	374,765	45.1	333.7	153.7	-53.9	7,428	3,639	-51
Chronic respiratory diseases	30-69 years	12,927	21,202	64	31.5	19.4	-38.5	1,227	792	-35.4
	All ages	36,881	72,316	96.1	50.3	30.1	-40.2	1,371	846	-38.3
Neoplasms	30-69 years	65,304	146,731	124.7	153	133.9	-12.5	5,258	4,620	-12.1
	All ages	111,628	276,459	147.7	126.1	111.4	-11.6	3,358	2,935	-12.6

PC: *Percentage of change*; CNCDs: Chronic noncommunicable diseases.

Table 2 shows the standardized rates of premature mortality from NCDs in both sexes for Brazil and its 27 federal units in 1990 and 2021. A reduction in mortality rates from NCDs, CVDs, and chronic respiratory diseases was observed in most states. The largest declines in CVD mortality rates were recorded in the Federal District (-72.9%) and Roraima (-72.1%). For diabetes and neoplasms, patterns varied across States. Mortality rates from neoplasms, for example, showed the largest reductions in Roraima (-52.3%) and the largest increase in Rio Grande do Norte (+35.6%), remaining relatively stable in most states.

**Table 2.** Standardized rates of premature mortality from chronic noncommunicable diseases and selected causes of death in Brazil and its federal units in 1990 and 2021.

Location	NCD			Cardiovascular disease			Chronic respiratory disease			Diabetes			Neoplasms		
	1990	2021	PC	1990	2021	PC	1990	2021	PC	1990	2021	PC	1990	2021	PC
Brazil	502.6	312.9	-37.7	286.6	135.7	-52.7	31.5	19.4	-38.5	31.5	24	-24	153	133.9	-12.5
Acre	366.2	303.2	-17.2	190.8	123.5	-35.3	30.9	27.5	-11.1	25	25.8	3.2	119.4	126.5	5.9
Alagoas	437.9	358.5	-18.1	259.3	180.9	-30.3	33.2	18.5	-44.2	39	43.6	11.8	106.5	115.6	8.5
Amapá	397.6	272.2	-31.5	206.5	108.5	-47.5	23.9	17.1	-28.6	23	24.6	7.1	144.3	122	-15.4
Amazon	379	309.9	-18.2	191.1	112.8	-41	22.1	16.9	-23.5	25.7	32.9	28.1	140.1	147.3	5.1
Bahia	381.6	291.2	-23.7	220.3	124.2	-43.6	24.3	17.5	-28	33.5	27.8	-16.9	103.5	121.7	17.6
Ceará	300.5	268.4	-10.7	154.2	118.6	-23.1	21.6	12.5	-42.1	18.3	16.7	-9	106.4	120.7	13.4
Federal District	481.2	187.4	-61	267.1	72.4	-72.9	25.3	9.8	-61.5	28.4	12.5	-56	160.2	92.8	-42.1
Espírito Santo	482.7	289.9	-39.9	288.1	129.2	-55.2	26.1	15.8	-39.4	26.2	20.5	-21.7	142.2	124.3	-12.6
Goiás	456.5	303.2	-33.6	260.4	134.9	-48.2	38	23.3	-38.6	23	22.3	-3.1	135	122.6	-9.2
Maranhão	414.9	357.5	-13.8	252.7	174.3	-31	20.9	16.6	-20.5	35.5	45.6	28.4	105.8	121	14.4
Mato Grosso	400.3	279.4	-30.2	229.2	120.1	-47.6	23.8	21.2	-11	25.2	25.7	2.1	122.2	112.4	-8
Mato Grosso do Sul	475.7	302.9	-36.3	288.1	139.9	-51.4	27.1	19.3	-29	23.2	18.9	-18.3	137.2	124.7	-9.1
Minas Gerais	510.4	292.9	-42.6	297.6	120.3	-59.6	34.3	18.7	-45.6	33	21.9	-33.6	145.5	132.1	-9.3
Paraná	562.7	311	-44.7	327.5	122.9	-62.5	42.5	22.4	-47.2	26.4	23	-12.9	166.4	142.8	-14.2
Paraíba	332.8	337.6	1.4	183.7	155	-15.6	20.1	16.5	-17.6	29.5	34.6	17.5	99.6	131.5	32
Pará	374.7	304.6	-18.7	216.4	135.3	-37.5	20	18.8	-5.8	21.9	32	46	116.5	118.5	1.7
Pernambuco	475.4	339.7	-28.5	286.5	159.9	-44.2	24.4	27.5	12.7	39.2	28.9	-26.2	125.3	123.4	-1.5
Piauí	331.1	275	-16.9	202.3	133.1	-34.2	18.7	11.6	-37.6	25	28.1	12.2	85.1	102.2	20.1
Rio Grande do Norte	286	295.7	3.4	150.4	130.2	-13.5	12.7	11.2	-11.4	30.6	29.2	-4.6	92.3	125.2	35.6
Rio Grande do Sul	565.4	321.7	-43.1	283.5	109	-61.6	49.6	27.1	-45.5	23.7	23.8	0.4	208.6	161.9	-22.4
Rio de Janeiro	680.4	367.2	-46	407.4	168.7	-58.6	33.9	19.2	-43.4	46.3	30.6	-33.9	192.7	148.7	-22.9
Rondônia	431	198.7	-53.9	249	84.2	-66.2	28	14.3	-48.9	28.8	18.3	-36.5	125.3	81.9	-34.6
Roraima	632.7	229.8	-63.7	348.5	97.3	-72.1	32.5	11.5	-64.8	54	26.8	-50.3	197.8	94.3	-52.3
Santa Catarina	514.9	291	-43.5	270.5	108.1	-60	47	21.2	-54.9	24.6	17.8	-27.4	172.8	143.9	-16.7
Sergipe	366.2	279	-23.8	189.3	119.9	-36.7	21.1	15.9	-24.7	40.6	30.6	-24.5	115.1	112.6	-2.2
São Paulo	556.9	327.7	-41.2	316.5	148.4	-53.1	31.1	19.2	-38.2	31.8	17.3	-45.6	177.6	142.8	-19.6
Tocantins	318.2	268.1	-15.8	193.2	128	-33.8	23.2	15	-35.6	22.1	28.5	29.1	79.7	96.6	21.2

PC: *Percentage of change*; NCDs: noncommunicable chronic diseases.

### Risk factors

Supplementary Table 1 shows the age-standardized SEV for RF related to NCDs in Brazil from 1990 to 2021. Between these years, the largest reductions in SEV for both sexes combined and all ages occurred for tobacco (-55.9%) and air pollution (-44.13%). In contrast, increases in SEV were observed for the following RFs: high BMI (+96.94%), high fasting blood glucose (+53.28%), and low physical activity (+39.68%). Similar results were found when analyzing the population aged 30 to 69 years.

### Projections for 2030 and 2050

**Figure 1.**
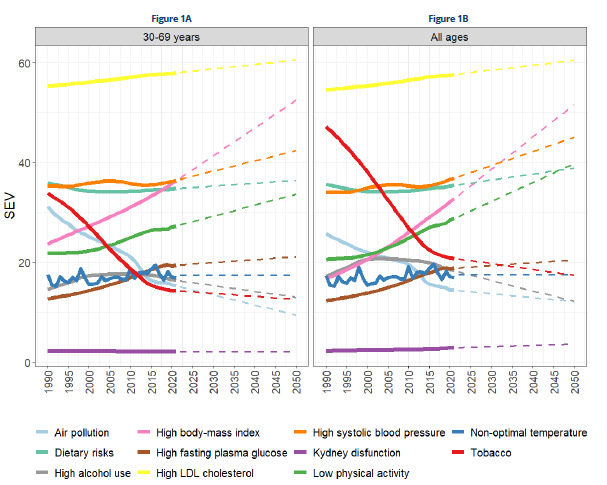
Trends and projections of risk factors, according to the *Summary Exposure* Value*, for Chronic Noncommunicable Diseases from 1990 to 2021 and projections until 2050.

When analyzing SEV projections until 2050, it was observed that high LDL cholesterol, blood pressure, fasting glucose, BMI, low physical activity, risks related to diet/nutrition, temperature, and renal function show an upward trend between 30 and 69 years of age and across all ages. Smoking, although declining at the beginning of the series, tended to stabilize between the ages of 30 and 69. Excessive alcohol consumption and exposure to air pollution showed a downward trend (Figure 1).

*The *Summary Exposure Value* (SEV) was considered.

**Figure 2.**
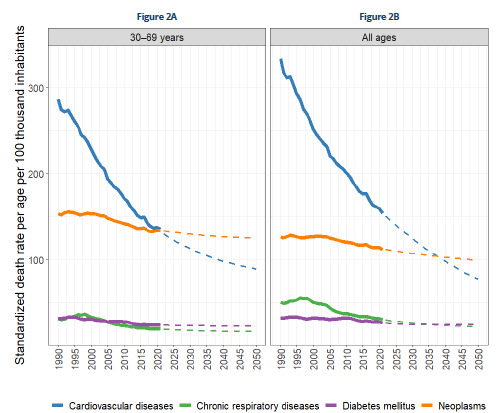
Trends in mortality rates for the four main groups of chronic noncommunicable diseases and projections for 2050, 1990-2021.

Figure 2 shows the trends and projections for mortality rates for the four main groups of CNCDs until 2050. Premature deaths from CVD continue to decline steadily and, from 2024/2025 onwards, these will be lower than those from neoplasms (the latter will reach the highest rates among all CNCDs in the coming decades). Mortality rates from chronic respiratory diseases and diabetes show more modest declines. When all age groups are considered, there will be declines in CVD, which remains in first place, followed by cancer and chronic respiratory diseases. In addition, there is an increase in diabetes rates, which will take the third place.

**Figure 3.**
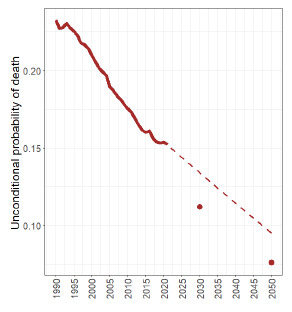
Mortality rates and probability of premature death from chronic noncommunicable diseases: trends (1990-2021) and projections (2030 and 2050), 1990-2021.

Figure 3 shows the trends in the probability of premature death from these diseases, with projections for 2030 and 2050. The global targets of reducing premature mortality from NCDs by 30% by 2030, based on data from 2015 to 2021, may not be achieved if the behavior observed during this period continues.

## DISCUSSION

The study pointed to significant decline in mortality from NCDs between 1990 and 2021, reflecting improved overall living conditions, expanded access to health care - such as the expansion of primary care^24^ , income distribution programs^25^ ^,^ ^26^, access to medicines, and improvements in medium- and high-complexity care - as well as the reduction of RFs, such as the prevalence of smoking and increased physical activity^4^. The fundamental role of SUS, which contributed to the improvement of various health indicators and outcomes^27^, should also be highlighted.

However, this decline has not been continuous over time; in 2016 and 2021, there was an increase in mortality rates from NCDs. These are attributed to deepening socioeconomic inequalities, fiscal austerity policies, and underfunding of SUS^5^ ^,^ ^28^. The increase observed in 2021 was attributed to the effects of the COVID-19 pandemic, which resulted in both a worsening of the population’s lifestyles - with an increase in sedentary lifestyles and feelings of sadness and loneliness, as well as a deterioration in diet - and an increase in mortality, especially from cardiovascular diseases. This scenario can be explained, in part, by the interruption or reduction in clinical follow-up and monitoring of people with NCDs during the pandemic, in addition to the possible effects of long COVID-19, which may have contributed to the worsening of health conditions and increased mortality from NCDs in the post-pandemic period^30^ ^,^ ^31^.

The largest reductions in CVD mortality rates can be explained by several factors, including the expansion of primary health care; income distribution programs; the reduction in smoking prevalence; and improvements in care, both through increased access to primary care services^13^ and expanded access to medications for the control of high blood pressure^32^.

The study showed that cancer will become the leading cause of premature death from NCDs in Brazil within a few years, signaling the continuation of the epidemiological transition process in the country. Neoplasms are already the second leading cause of death in most countries^21^. In Uruguay and Argentina, cancer is the leading cause of death^33^. In Brazil, studies point to an increasing trend in the incidence of some types of cancer between 1990 and 2015, such as lung cancer in women (+20.7%) and colon and rectal cancer in men (+29.5%)^34^. This change reflects alterations in patterns of exposure to risk factors, therapeutic advances, and early screening and diagnosis systems^35^.

Diabetes is associated with increased mortality from cardiovascular diseases, with systemic effects that impact various organs and functions of the body^23^. There is consistent evidence that the growth in the prevalence of diabetes is strongly associated with the increase in obesity in the population^23^.

The significant reduction in mortality rates from chronic respiratory diseases can be attributed, in large part, to the decline in the prevalence of smoking^4^ ^,^ ^36^. Other factors that may have contributed to this trend include increased access to health services, especially with the expansion of Primary Health Care (PHC) in the country^37^.

According to the analysis by federal units, the patterns show some similarity between States. As for diabetes and neoplasms, there is a trend of increase or stabilization in most States. It is noteworthy that the States in the North and Northeast regions tend to show smaller reductions or even increases in rates, which may be related to worse living conditions, but also to possible limitations in the quality of information in mortality registration systems, especially in previous years^13^ ^,^ ^38^ ^,^ ^39^.

### Risk factors

The results show a significant reduction in the main RFs, especially behavioral ones, with an emphasis on smoking. Evidence indicates that the control of risk factors and NCDs is more effective when regulatory measures are implemented by the State^2^ ^,^ ^4^ ^,^ ^40^ . In the case of tobacco, several regulatory actions have been adopted, such as accession to the Framework Convention on Tobacco Control (FCTC) in 2006; and Presidential Decree No. 8,262, of 2014, which expanded warnings on cigarette packs and regulated smoke-free environments and taxation on tobacco products^4^ ^,^ ^13^ . However, since 2016, the country has experienced a weakening of public policies to control smoking, with stagnation in pricing and taxation policies, as well as growing interference by the tobacco industry in the political and legal spheres, highlighting the influence of commercial determinants on health^8^ and resulting in poorer policy performance^36^ ^,^ ^41^ ^,^ ^42^.

Metabolic risk factors project high risks in the future, posing a threat to the health of the population in the coming years. In the last decade, Brazil has implemented important policies to strengthen PHC, such as the creation of the *Mais Médicos* (More Doctors) Program, expansion of free access to medicines for NCDs, health promotion actions, and income transfer programs^6^ ^,^ ^32^.

 Obesity is a serious public health problem in Brazil, with a tendency to increase in prevalence among the adult population. It is estimated that approximately one-third of adults in Brazilian State capitals are obese and about 60% are overweight^11^. This rapid growth reinforces the need to expand access to obesity treatment; encourage regular physical activity; promote exclusive breastfeeding; and advance fiscal policies, such as increasing taxation on ultra-processed foods. These measures aim to strengthen access to healthy eating throughout life, in accordance with the guidelines of the Food Guide for the Brazilian Population.

Inadequate nutrition is projected to increase the SEV by 2050. Evidence shows an increase in ultra-processed foods and a reduction in the consumption of fresh foods, such as fruits, vegetables, and beans, as a result of changes in eating patterns, the economic crisis, and the adoption of austerity policies since 2016^5^ ^,^ ^44^.

The “best evidence” indicated by the WHO for the prevention and control of NCDs includes regulatory and health promotion measures, such as taxation of beverages, tobacco, and ultra-processed foods, as well as a ban on the marketing of products harmful to health^45^. Therefore, they depend on investment in public policies and political decisions to advance the proposed agendas, including confronting the interests of industrial and commercial lobby^45^.

### Projections for 2030 and 2050

The study indicates that projections of NCD mortality rates for 2030 and 2050 signal the possibility of failure to meet the established reduction targets. This scenario reinforces the need for continuous monitoring to identify possible changes in disease patterns, their determinants, and risk factors, and to adjust coping strategies in a timely manner over the coming years.

#### What are the global and national challenges to achieving the 2050 targets?

Investment in health promotion and NCD prevention actions can result in significant advances, provided there is political will and effective implementation of preventive measures^10^. A concrete example is the global reduction in smoking by about 25% in most countries since the adoption of the FCTC in 2007^10^ ^,^ ^46^. In China, levels of fine particulate matter (PM2.5) in the air have been reduced by almost 50% in the last decade, demonstrating the effectiveness of strict environmental policies^47^.

In this context, the measures approved in Brazil regarding tax reform on tobacco and soft drinks represent a strategic opportunity to strengthen fiscal measures on products that are harmful to health, with a potential impact not only on mortality projections until 2050, but also on the sustainability of NCD prevention and control in the long term.

### Study limitations

Among the limitations of the study, the projections for 2030 and 2050 were based on trends observed in previous years, assuming the continuity of past patterns as a parameter for the future. However, the future is uncertain and may include unpredictable events, as demonstrated by the COVID-19 pandemic. Incomplete data sources, such as the SIM, have made progress in coverage and improvement of record quality, although underreporting and a high proportion of garbage codes persist^19^. Additionally, social determinants are not directly incorporated into causal analyses^48^.

The study points out that global targets may not be achieved, raising an alert to the urgent need to strengthen public policies aimed at the prevention and control of CNCDs. It highlights the importance of intensifying intersectoral actions and strategies for health promotion, early diagnosis, timely access to treatment, and continuous monitoring - especially in the context of PHC.

### Supplementary Materials

Table 1

## References

[1] World Health Organization (2013). Global action plan for the prevention and control of noncommunicable diseases 2013-2020.

[2] Malta DC, Duncan BB, Schmidt MI, Teixeira R, Ribeiro ALP, Felisbino-Mendes MS (2020). Trends in mortality due to non-communicable diseases in the Brazilian adult population: national and subnational estimates and projections for 2030. Popul Health Metr.

[3] World Health Organization (2023). Commercial determinants of health.

[4] Malta DC, Flor LS, Machado IE, Felisbino-Mendes MS, Brant LCC, Ribeiro ALP (2020). Trends in prevalence and mortality burden attributable to smoking, Brazil and federated units, 1990 and 2017. Popul Health Metr.

[5] Malta DC, Duncan BB, Barros AMB, Katikireddi SV, Souza FM, Silva AG (2018). Medidas de austeridade fiscal comprometem metas de controle de doenças não transmissíveis no Brasil. Ciênc Saúde Colet.

[6] Malta DC, Gomes CS, Stopa SR, Andrade FMD, Prates EJS, Oliveira PPV (2022). Inequalities in health care and access to health services among adults with self-reported arterial hypertension: Brazilian National Health Survey. Cad Saude Publica.

[7] Pearce N, Ebrahim S, McKee M, Lamptey P, Barreto ML, Matheson D (2014). The road to 25×25: how can the five-target strategy reach its goal?. Lancet Glob Health.

[8] Gilmore AB, Fabbri A, Baum F, Bertscher A, Bondy K, Chang HJ (2023). Defining and conceptualising the commercial determinants of health. Lancet.

[9] Organização das Nações Unidas (2015). ransformando nosso mundo: a Agenda 2030 para o Desenvolvimento Sustentável.

[10] Alleyne G, Coll-Seck AM, Frieden TR, Tufton C (2025). Fourth time a charm? -How to make the UN high-level meeting on noncommunicable diseases effective. JAMA.

[11] Malta DC, Gomes CS, Prates EJS, Bernal RTI (2023). Changes in chronic diseases and risk and protective factors before and after the third wave of COVID-19 in Brazil. Ciênc Saúde Coletiva.

[12] Malta DC, Gomes CS, Silva AG, Cardoso LSM, Barros MBA, Lima MG (2021). Uso dos serviços de saúde e adesão ao distanciamento social por adultos com doenças crônicas na pandemia de COVID-19, Brasil, 2020. Ciênc Saúde Coletiva.

[13] Malta DC, Gomes CS, Veloso GA, Teixeira RA, Mendes MSF, Brant LCC (2024). Noncommunicable disease burden in Brazil and its states from 1990 to 2021, with projections for 2030. Public Health.

[14] Jamison DT, Summers LH, Chang AY, Karlsson O, Mao W, Norheim OF (2024). Global health 2050: the path to halving premature death by mid-century. Lancet.

[15] Fundação Oswaldo Cruz (2022). O projeto - saúde amanhã.

[16] Fundação Oswaldo Cruz As seis transformações: TWI2050.

[17] Brasil (2022). Sistema de Informações sobre Mortalidade (SIM). Departamento de Informática do Sistema Único de Saúde.

[18] Naghavi M, Makela S, Foreman K, O’Brien J, Pourmalek F, Lozano R (2010). Algorithms for enhancing public health utility of national causes-of-death data. Popul Health Metr.

[19] Teixeira RA, Naghavi M, Guimarães MDC, Ishitani LH, França EB (2019). Quality of cause-of-death data in Brazil: Garbage codes among registered deaths in 2000 and 2015. Rev Bras Epidemiol.

[20] Malta DC, Felisbino-Mendes MS, Machado IE, Passos VMA, Abreu DMX, Ishitani LH (2017). Fatores de risco relacionados à carga global de doença do Brasil e Unidades Federadas, 2015. Rev Bras Epidemiol.

[21] Fitzmaurice C, Abate D, Abbasi N, Abbastabar H, Abd-Allah F, Global Burden of Disease Cancer Collaboration (2019). Global, Regional, and National Cancer Incidence, mortality, years of life lost, years lived with disability, and disability-adjusted life-years for 29 cancer groups, 1990 to 2017: a systematic analysis for the Global Burden of Disease Study. JAMA Oncol.

[22] GBD 2015 Risk Factors Collaborators (2016). Global, regional, and national comparative risk assessment of 79 behavioural, environmental and occupational, and metabolic risks or clusters of risks, 1990-2015: a systematic analysis for the Global Burden of Disease Study 2015. Lancet.

[23] GBD 2021 Diabetes Collaborators (2023). Global, regional, and national burden of diabetes from 1990 to 2021, with projections of prevalence to 2050: a systematic analysis for the Global Burden of Disease Study 2021. Lancet.

[24] Rasella D, Harhay MO, Pamponet ML, Aquino R, Barreto ML (2014). Impact of primary health care on mortality from heart and cerebrovascular diseases in Brazil: a nationwide analysis of longitudinal data. BMJ.

[25] Pescarini JM, Campbell D, Amorim LD, Falcão IR, Ferreira AJF, Allik M (2022). Impact of Brazil’s Bolsa Família Programme on cardiovascular and all-cause mortality: a natural experiment study using the 100 Million Brazilian Cohort. Int J Epidemiol.

[26] Guimarães JMN, Pescarini JM, Sousa JF, Ferreira A, Almeida MCC, Gabrielli L (2024). Income segregation, conditional cash transfers, and breast cancer mortality among women in Brazil. JAMA Netw Open.

[27] Martins TCF, Silva JHC, Máximo GC, Guimarães RM (2021). Transição da morbimortalidade no Brasil: um desafio aos 30 anos de SUS. Ciên Saúde Coletiva.

[28] Castro MC, Massuda A, Almeida G, Menezes-Filho NA, Andrade MV, Noronha KVMS (2019). Brazil’s unified health system: the first 30 years and prospects for the future. Lancet.

[29] Brant LCC, Nascimento BR, Teixeira RA, Lopes MACQ, Malta DC, Oliveira GMM (2020). Excess of cardiovascular deaths during the COVID-19 pandemic in Brazilian capital cities. Heart.

[30] Shu J, Jin W (2023). Prioritizing non-communicable diseases in the post-pandemic era based on a comprehensive analysis of the GBD 2019 from 1990 to 2019. Sci Rep.

[31] Malta DC, Gomes CS, Barros MBA, Lima MG, Almeida WS, Sá ACMGN (2021). Doenças crônicas não transmissíveis e mudanças nos estilos de vida durante a pandemia de COVID-19 no Brasil. Rev Bras Epidemiol.

[32] Malta DC, Bernal RTI, Prates EJS, Vasconcelos NM, Gomes CS, Stopa SR (2022). Hipertensão arterial autorreferida, uso de serviços de saúde e orientações para o cuidado na população brasileira: Pesquisa Nacional de Saúde, 2019. Epidemiol Serv Saúde.

[33] Malta DC, Gomes CS, Veloso GA, Dias de Andrade FM, Souza JB, Freitas PC (2023). Burden of non-communicable diseases and the achievement of the sustainable development goals in 2030 in Mercosur countries. Public Health.

[34] Guerra MR, Bustamante-Teixeira MT, Corrêa CSL, Abreu DMX, Curado MP, Mooney M (2017). Magnitude e variação da carga da mortalidade por câncer no Brasil e Unidades da Federação, 1990 e 2015. Rev Bras Epidemiol.

[35] Rache B, Rocha R, Medeiros LA, Okada LM, Ferrari G, Zeng H (2024). Transition towards cancer mortality predominance over cardiovascular disease mortality in Brazil, 2000-2019: a population-based study. Lancet Reg Health Am.

[36] Malta DC, Gomes CS, Andrade FMD, Prates EJS, Alves FTA, Oliveira PPV (2021). Tobacco use, cessation, secondhand smoke and exposure to media about tobacco in Brazil: results of the National Health Survey 2013 and 2019. Rev Bras Epidemiol.

[37] Santos FM, Macieira C, Machado ATGM, Borde EMS, Jorge AO, Gomes BA (2023). Associação entre internações por condições sensíveis e qualidade da atenção primária. Rev Saude Publica.

[38] Ishitani LH, Teixeira RA, Abreu DMX, Paixão LMMM, França EB (2017). Qualidade da informação das estatísticas de mortalidade: códigos garbage declarados como causas de morte em Belo Horizonte, 2011-2013. Rev Bras Epidemiol.

[39] Malta DC, Teixeira RA, Cardoso LSM, Souza JB, Bernal RTI, Pinheiro PC (2023). Premature mortality due to noncommunicable diseases in Brazilian capitals: redistribution of garbage causes and evolution by social deprivation strata. Rev Bras Epidemiol.

[40] Malta DC, Felisbino-Mendes MS, Machado IE, Veloso GA, Gomes CS, Brant LCC (2022). Burden of disease attributable to risk factors in Brazil: an analysis of national and subnational estimates from the 2019 Global Burden of Disease study. Rev Soc Bras Med Trop.

[41] Meirelles RHS (2023). Editorial: os avanços do controle do tabagismo no Brasil. Physis: Revista de Saúde Coletiva.

[42] Cavalcante TM, Pinho MCM, Perez CA, Teixeira APL, Mendes FL, Vargas RR (2017). Brasil: balanço da Política Nacional de Controle do Tabaco na última década e dilemas. Cad Saúde Pública.

[43] Jaime PC, Delmuè DCC, Campello T, Silva DO, Santos LMP (2018). Um olhar sobre a agenda de alimentação e nutrição nos trinta anos do Sistema Único de Saúde. Ciênc Saúde Coletiva.

[44] Silva AG, Teixeira RA, Prates EJS, Malta DC (2021). Monitoramento e projeções das metas de fatores de risco e proteção para o enfrentamento das doenças crônicas não transmissíveis nas capitais brasileiras. Ciênc Saúde Coletiva.

[45] World Health Organization (2024). Tackling NCDs: best buys and other recommended interventions for the prevention and control of noncommunicable diseases.

[46] World Health Organization (2023). Countdown to 2023: WHO 5-year milestone report on global trans fat elimination 2023.

[47] Greenstone M, Ganguly T, Hasenkopf C, Sharma N, Gautam H (2024). Air quality life index annual update.

[48] Pearce N, Ebrahim S, McKee M, Lamptey P, Barreto ML, Matheson D (2015). Global prevention and control of NCDs: Limitations of the standard approach. J Public Health Policy.

