# Supplementary material for: Noncommunicable chronic diseases and health challenges in 2050
Source: Rev Bras Epidemiol. 2026 Mar 16;29:e260011. doi: 10.1590/1980-549720260011 (PMC12995342; doi:10.1590/1980-549720260011)
Supplement: Table 1 [file 1980-5497-rbepid-29-e260011-sppl.pdf]

Tabela 1 Suplementar - *Summary Exposure Value* (SEV) para fatores de risco selecionados (Nível 2), em 1990 e 2021 e percentual (%) de mudança no período, GBD Brasil, 2019 - 2021.

**Fator de Risco**	**Faixa Etaria**	**1990**	**1995**	**2000**	**2005**	**2010**	**2015**	**2020**	**2021**	**PC 1990-2021**
Colesterol LDL alto	30-69 anos	55,31	55,68	56,09	56,52	57,05	57,58	57,86	57,95	4,76
	Todas as idades	54,55	54,94	55,38	55,86	56,48	57,13	57,52	57,62	5,62
Função renal prejudicada	30-69 anos	2,17	2,20	2,19	2,16	2,13	2,09	2,11	2,10	-2,88
	Todas as idades	2,24	2,32	2,40	2,46	2,53	2,62	2,80	2,82	25,59
Glicose plasmática em jejum elevada	30-69 anos	12,65	13,37	14,31	15,44	16,83	18,48	19,24	19,38	53,22
	Todas as idades	12,27	12,95	13,83	14,91	16,32	17,89	18,70	18,81	53,28
Poluição do ar	30-69 anos	31,18	27,66	25,16	23,32	21,06	16,30	15,46	15,26	-51,06
	Todas as idades	25,76	23,79	21,99	20,80	19,53	15,64	14,47	14,39	-44,13
Pouca atividade física	30-69 anos	21,80	21,87	22,24	23,38	24,92	26,38	27,06	27,22	24,86
	Todas as idades	20,58	20,82	21,46	22,88	24,81	26,94	28,43	28,74	39,68
Pressão arterial sistólica elevada	30-69 anos	35,34	35,20	35,86	36,32	35,79	35,49	36,21	36,41	3,03
	Todas as idades	34,04	34,01	34,88	35,57	35,26	35,33	36,56	36,84	8,23
Riscos dietéticos	30-69 anos	35,90	34,97	34,26	34,10	34,21	34,47	34,68	34,74	-3,24
	Todas as idades	35,58	34,74	34,17	34,09	34,29	34,77	35,29	35,41	-0,49
Tabagismo	30-69 anos	33,78	31,06	27,20	22,65	18,72	15,39	14,36	14,30	-57,67
	Todas as idades	47,16	43,09	38,10	32,42	26,86	22,42	20,89	20,77	-55,95
Temperatura	30-69 anos	17,51	15,90	15,60	16,71	18,85	18,55	17,02	16,91	-3,41
	Todas as idades	17,29	15,83	15,43	16,90	19,04	18,80	17,06	16,92	-2,15
Uso excessivo de álcool	30-69 anos	14,58	15,89	17,28	17,59	17,65	17,40	16,51	16,39	12,43
	Todas as idades	17,24	18,91	20,59	20,67	20,42	19,86	18,49	18,28	6,05
Índice de massa corporal elevado	30-69 anos	23,65	25,48	27,22	29,07	31,02	33,25	35,73	36,29	53,45
	Todas as idades	16,63	18,49	20,57	23,10	25,98	29,01	32,09	32,74	96,94
