# Supplementary material for: Noncommunicable chronic diseases and health challenges in 2050
Source: Rev Bras Epidemiol. 2026 Mar 16;29:e260011. doi: 10.1590/1980-549720260011 (PMC12995342; doi:10.1590/1980-549720260011)
Supplement: Translation

# Translation: As doenças crônicas não transmissíveis e os desafios à saúde em 2050

**DOI:** 10.1590/1980-549720260011.2

**Authors:** Deborah Carvalho Malta, Guilherme Augusto Veloso, Crizian Saar Gomes, Maurício Lima Barreto

**Affiliations:** I Universidade Federal de Minas Gerais, Escola de Enfermagem, Departamento de Enfermagem Materno-infantil e Saúde Pública - Belo Horizonte (MG), Brasil.; II Universidade Federal Fluminense - Niterói (RJ), Brasil.; III Universidade Federal de Minas Gerais, Faculdade de Medicina, Programa de Pós-Graduação em Saúde Pública, - Belo Horizonte (MG), Brasil.; IV Fundação Oswaldo Cruz, Centro de Integração de Dados e Conhecimentos para Saúde, Instituto Gonçalo Moniz - Salvador (BA), Brasil.

**Keywords:** Doenças crônicas não transmissíveis, Fatores de risco, Carga global da doença, Brasil

## Abstract

**Objetivos:**

O estudo visa analisar a carga de doenças relacionadas às Doenças Crônicas não Transmissíveis (DCNT) e aos seus Fatores de Risco (FR) no período de 1990 a 2021; e projetar a evolução de DCNT e FR para os anos de 2030 e 2050.

**Métodos:**

Foram analisadas estimativas de fatores de risco e de mortalidade por DCNT no Brasil e em suas unidades da federação, com base nos dados do Estudo Global de Doenças.

**Resultados:**

Observou-se uma redução de 37,7% nas taxas de mortalidade prematura por DCNT e de 34% em Anos de Vida Ajustados por Incapacidade (DALYs) entre 1990 e 2021. As projeções para 2050 indicam que a mortalidade por doenças cardiovasculares continuará em queda, e que estas serão suplantadas pelas neoplasias. As taxas de mortalidade por diabetes tendem a aumentar, enquanto as doenças respiratórias crônicas apresentam tendência de redução. Em 2021, os principais FR associados às mortes prematuras por DCNT foram: pressão arterial elevada, tabagismo, alimentação inadequada, Índice de Massa Corporal (IMC) elevado e glicose em jejum elevada. As projeções até 2050 apontam aumento nos fatores de risco obesidade, pressão arterial elevada, glicose elevada, inatividade física e dieta inadequada - o que pode comprometer o alcance das metas de redução da mortalidade prematura por DCNT estabelecidas para 2050.

**Conclusão:**

O estudo aponta que as metas globais podem não ser alcançadas, acendendo um alerta para a necessidade urgente de fortalecimento das políticas públicas voltadas à prevenção e ao controle das DCNT.

## INTRODUÇÃO

As Doenças Crônicas não Transmissíveis (DCNT) constituem a principal causa de morbimortalidade global, resultando em mortes prematuras, incapacidades, perda da qualidade de vida, impactos econômicos e sobrecarga dos sistemas de saúde^1^ ^,^ ^2^. Estima-se que as DCNT sejam responsáveis por 75% da mortalidade global^3^.

Os quatro principais grupos de DCNT - doenças cardiovasculares (DCV), câncer, doenças respiratórias crônicas e diabetes - compartilham Fatores de Risco (FR) comportamentais comuns e modificáveis, como o tabagismo, consumo abusivo de bebidas alcoólicas, a inatividade física e alimentação inadequada^4^ ^,^ ^5^. Além desses, destacam-se também os fatores metabólicos, como hipertensão arterial, obesidade, colesterol elevado e glicemia elevada^2^ ^,^ ^5^ ^,^ ^6^. Na rede de causalidade das DCNT e de seus FR, atuam fortemente os determinantes socioeconômicos^1^ ^,^ ^7^ ^,^ ^8^.

A Agenda 2030 para o Desenvolvimento Sustentável estabeleceu como meta a redução, em um terço, da “probabilidade incondicional de morte prematura por DCNT”^9^. Até o momento, apenas 19 países - representando somente 2,7% da população mundial - estão no caminho para alcançar esse objetivo. No ritmo atual, a meta levaria aproximadamente 45 anos para ser atingida^10^.

No Brasil, estudos indicam que as taxas de mortalidade por DCNT apresentaram tendência de queda entre 2010 e 2015. No entanto, esse progresso foi interrompido a partir de 2016 e se agravou durante a pandemia de COVID-19, comprometendo a possibilidade de alcançar a meta de redução de 30% estabelecida pelo Plano de Enfrentamento das Doenças e Agravos não Transmissíveis (Plano de DANT)^2^ ^,^ ^11^ ^,^ ^12^ ^,^ ^13^.

Diante dos desafios globais persistentes, novos compromissos têm sido propostos. Em 2024, a *Lancet Commission* lançou a meta de reduzir em 50% a probabilidade de morte prematura por doenças selecionadas até 2050 - uma proposta que ficou conhecida como “50 por 50”. Essa meta poderá ser atingida por meio do enfrentamento de 15 condições prioritárias - sendo 8 relacionadas a doenças infecciosas e à saúde materna; e 7, às DCNT e lesões^14^.

As principais propostas para atingir as metas em 2050 seriam

a) concentrar recursos em condições prioritárias, incluindo as DCNT, com vistas a maximizar o impacto na redução da mortalidade prematura;
b) ampliar o financiamento global em saúde;
c) controle do tabaco como estratégia prioritária, com elevação de impostos, proibição de marketing e outros;
d) redução da pobreza e das desigualdades sociais;
e) melhoria do acesso a atenção primária e medicamentos essenciais;
f) promoção de alimentação saudável, atividade física e ambientes saudáveis.

Nesse contexto, destaca-se a *Iniciativa Brasil Saúde Amanhã*, da Fundação Oswaldo Cruz (Fiocruz), que parte da pergunta norteadora: “*Qual o futuro da saúde no Brasil?*”. A iniciativa articula uma rede multidisciplinar de pesquisa voltada à investigação, prospecção estratégica e formulação de políticas públicas para o setor da saúde, fortalecendo o Sistema Único de Saúde (SUS)^15^ ^,^ ^16^.

Este estudo se insere nesse escopo e tem como objetivos: analisar a carga de doenças relacionadas às DCNT e os seus fatores de risco no período de 1990 a 2021; e projetar a evolução de DCNT e FR para os anos de 2030 e 2050.

## MÉTODOS

Este estudo analisou as estimativas de fatores de risco e de mortalidade por DCNT no Brasil e em suas unidades da federação, com base nos dados do Estudo Global de Doenças (*Global Burden of Disease* - GBD) 2021, do Instituto de Métricas e Avaliação em Saúde (Institute for Health Metrics and Evaluation - IHME, em inglês), disponíveis em: http://ghdx.healthdata.org/.

### Mortalidade

As estimativas de mortalidade do GBD são baseadas nos dados do Sistema de Informações sobre Mortalidade (SIM), do Ministério da Saúde^17^, com ajustes para os chamados *garbage codes*, por meio de algoritmos de redistribuição das causas básicas de morte, considerando idade, sexo e ano^18^ ^,^ ^19^.

Foram calculadas as probabilidades incondicionais de morte prematura por DCNT. Para cada faixa etária (𝑥,𝑥+5), a probabilidade de morte durante o intervalo foi estimada com base na taxa de mortalidade específica por idade, denotada por *m*_*x*_ , utilizando a aproximação $q_{x}=\frac{{5m}_{x}}{1+{2,5m}_{x}}$. A probabilidade incondicional de morte foi estimada como:

$$P\left(30\leq idade\;de\;morte\leq 69\right)=1-\prod_{x=30}^{65}\left(1-p_{x}\right)$$

As métricas utilizadas para analisar a carga das DCNT no Brasil foram: número absoluto de óbitos, taxas de mortalidade padronizadas por idade e anos de vida perdidos por morte prematura e Ajustados por Incapacidade (DALYs). Essas métricas foram apresentadas para todas as faixas etárias e para mortes prematuras, entre 30 e 69 anos de idade, segundo o Plano Global da Organização Mundial da Saúde (OMS)^1^. As análises contemplaram o total de doenças e os 4 principais grupos de DCNT no Brasil nos anos de 1990 e 2021, além da variação percentual (Percentual de Mudança - PC) no período.

Para verificar as desigualdades subnacionais, analisaram-se as taxas de mortalidade prematura por DCNT para os 26 estados brasileiros e o Distrito Federal em 1990 e 2021.

### Fatores de risco

As principais fontes de dados sobre FR para a população brasileira utilizadas pelo GBD foram a Pesquisa Nacional de Saúde (PNS), o Sistema de Vigilância por Inquérito Telefônico de Fatores de Risco e Proteção para Doenças Crônicas (Vigitel) e a Pesquisa Nacional de Saúde do Escolar (PeNSE)^13^ ^,^ ^20^.

O estudo GBD utiliza uma lista hierárquica de fatores de risco organizada em quatro níveis, que vão de grupos amplos (metabólicos, comportamentais e ambientais) a mais detalhados, totalizando 87 fatores analisados em 2021^21^.

Em relação aos FR, analisou-se o *Summary Exposure Value* (SEV). O SEV representa a prevalência da exposição ponderada pelo risco relativo, variando de 0% a 100%, em que 0% indica ausência total de exposição e 100% corresponde ao nível máximo de exposição observada na população. A redução do SEV indica diminuição da exposição populacional ao fator de risco, enquanto seu aumento sugere o oposto. Mais informações sobre o SEV estão disponíveis em outras publicações^20^ ^,^ ^22^.

### Projeções para 2050

#### Fatores de risco

Foram analisadas as tendências do SEV para fatores de risco relacionados às DCNT entre 1990 e 2021, incluindo: tabagismo, alimentação inadequada, baixa atividade física, consumo excessivo de álcool, pressão arterial elevada, colesterol elevado, função renal prejudicada, glicemia de jejum elevada, Índice de Massa Corporal (IMC) elevado, poluição do ar e temperatura. Com base na evolução observada nesse período, foram calculadas projeções desses indicadores até o ano de 2050.

#### Projeções de mortalidade

Foram calculadas as projeções das taxas de mortalidade prematura e de mortalidade em todas as idades por DCNT e por seus quatro principais grupos, assim como a probabilidade de morte prematura para os anos de 2030 e 2050. Esses resultados foram comparados às metas dos Objetivos de Desenvolvimento Sustentável (ODS), pactuadas em 2015, que estimam a redução de 30% da probabilidade incondicional de morte prematura por DCNT^23^. Foram, ainda, feitas projeções para comparar com a meta proposta pela *Lancet Commission* (redução de 50% até 2050)^14^. Para os cálculos das projeções, foram utilizados como referência os valores observados nos anos de 2015 a 2021.

Foi utilizado um modelo de ETS. O termo refere-se à classe de modelos que combinam três componentes fundamentais: Erro (E), Tendência (T) e Sazonalidade (S); do tipo: Aditivo (A), tendência Aditiva (A) e ausência de sazonalidade (N - de *None*) (AAN). Esse modelo é especialmente indicado para séries temporais que exibem tendência, mas não apresentam padrões sazonais. Além disso, foi utilizada uma tendência amortecida, que introduz um fator de amortecimento para reduzir gradualmente a influência da tendência nas projeções de longo prazo.

As análises foram conduzidas no *RStudio* (*RStudio Team*, 2019) e as figuras, produzidas utilizando o pacote *ggplot214*.

#### Considerações éticas

O projeto GBD-Brasil foi aprovado pelo Comitê de Ética em Pesquisa da Universidade Federal de Minas Gerais (UFMG), registrado sob o número do Projeto CAAE - 62803316.7.0000.5149.

## Declaração de disponibilidade de dados

Todos dados que dão suporte aos resultados deste estudo estão disponíveis em: http://ghdx.healthdata.org

## RESULTADOS

### Mortalidade

A Tabela 1 apresenta a carga de mortalidade e DALY por DCNT no Brasil em 1990 e 2021. As taxas de mortalidade prematura por todas as causas reduziram-se em 7,1%. No mesmo período, observou-se uma redução de 37,7% nas taxas de mortalidade prematura por DCNT e de 34% nas taxas de DALYs. Entre 1990 e 2021, as taxas de mortalidade prematura por DCV, doenças respiratórias crônicas, diabetes mellitus e neoplasias diminuíram 52,7%, 38,5%, 24% e 12,5%, respectivamente. As taxas de DALYs apresentaram reduções semelhantes (Tabela 1).

**Tabela 1.** Número de óbitos e taxas padronizadas de mortalidade e de DALYs na população entre 30 e 69 anos e em todas as idades, de ambos os sexos, no Brasil, em 1990 e 2021.

Causa de morte	Faixa etária	Número de óbitos			Taxa de mortalidade padronizada (por 100 mil)			Taxa de DALYs padronizada (por 100 mil)		
		1990	2021	PC	1990	2021	PC	1990	2021	PC
Todas as causas	30-69 anos	350.153	804.109	129,6	792,1	735,8	-7,1	44.647	42.524	-4,8
	Todas as idades	875.709	1.796.921	105,2	926,4	742,9	-19,8	43.050	33.056	-23,2
DCNT	30-69 anos	212.908	342.930	61,1	502,6	312,9	-37,7	18.103	11.942	-34
	Todas as idades	433.065	788.379	82	542,2	321,8	-40,7	13.261	8.500	-35,9
Diabetes mellitus	30-69 anos	13.191	26.289	99,3	31,5	24	-24	1.656	1.627	-1,7
	Todas as idades	26.319	64.839	146,4	32,1	26,5	-17,5	1.104	1.080	-2,2
Doenças cardiovasculares	30-69 anos	121.486	148.709	22,4	286,7	135,7	-52,7	9.962	4.902	-50,8
	Todas as idades	258.237	374.765	45,1	333,7	153,7	-53,9	7.428	3.639	-51
Doenças respiratórias crônicas	30-69 anos	12.927	21.202	64	31,5	19,4	-38,5	1.227	792	-35,4
	Todas as idades	36.881	72.316	96,1	50,3	30,1	-40,2	1.371	846	-38,3
Neoplasias	30-69 anos	65.304	146.731	124,7	153	133,9	-12,5	5.258	4.620	-12,1
	Todas as idades	111.628	276.459	147,7	126,1	111,4	-11,6	3.358	2.935	-12,6

PC: *Percentage of change*; DCNT: Doenças Crônicas não Transmissíveis.

A Tabela 2 apresenta as taxas padronizadas de mortalidade prematura por DCNT, em ambos os sexos, para o Brasil e as 27 unidades federativas, nos anos de 1990 e 2021. Observou-se uma redução nas taxas de mortalidade por DCNT, DCV e doenças respiratórias crônicas na maioria dos estados. As maiores quedas nas taxas de mortalidade por DCV foram registradas no Distrito Federal (-72,9%) e no Paraná (-62,5%). Para diabetes e neoplasias, os padrões variaram entre os estados. As taxas de mortalidade por neoplasias, por exemplo, apresentaram as maiores reduções no Roraima (-52,3%) e o maior aumento no Rio Grande do Norte (+35,6%), mantendo-se relativamente estável na maioria das unidades federativas.

**Tabela 2.** Taxas padronizadas de mortalidade prematura por Doenças Crônicas não Transmissíveis e causas selecionadas de morte no Brasil e em suas unidades federativas, em 1990 e 2021.

Localização	DCNT			Doença cardiovascular			Doença respiratória crônica			Diabetes mellitus			Neoplasias		
	1990	2021	PC	1990	2021	PC	1990	2021	PC	1990	2021	PC	1990	2021	PC
Brasil	502,6	312,9	-37,7	286,6	135,7	-52,7	31,5	19,4	-38,5	31,5	24	-24	153	133,9	-12,5
Acre	366,2	303,2	-17,2	190,8	123,5	-35,3	30,9	27,5	-11,1	25	25,8	3,2	119,4	126,5	5,9
Alagoas	437,9	358,5	-18,1	259,3	180,9	-30,3	33,2	18,5	-44,2	39	43,6	11,8	106,5	115,6	8,5
Amapá	397,6	272,2	-31,5	206,5	108,5	-47,5	23,9	17,1	-28,6	23	24,6	7,1	144,3	122	-15,4
Amazonas	379	309,9	-18,2	191,1	112,8	-41	22,1	16,9	-23,5	25,7	32,9	28,1	140,1	147,3	5,1
Bahia	381,6	291,2	-23,7	220,3	124,2	-43,6	24,3	17,5	-28	33,5	27,8	-16,9	103,5	121,7	17,6
Ceará	300,5	268,4	-10,7	154,2	118,6	-23,1	21,6	12,5	-42,1	18,3	16,7	-9	106,4	120,7	13,4
Distrito Federal	481,2	187,4	-61	267,1	72,4	-72,9	25,3	9,8	-61,5	28,4	12,5	-56	160,2	92,8	-42,1
Espírito Santo	482,7	289,9	-39,9	288,1	129,2	-55,2	26,1	15,8	-39,4	26,2	20,5	-21,7	142,2	124,3	-12,6
Goiás	456,5	303,2	-33,6	260,4	134,9	-48,2	38	23,3	-38,6	23	22,3	-3,1	135	122,6	-9,2
Maranhão	414,9	357,5	-13,8	252,7	174,3	-31	20,9	16,6	-20,5	35,5	45,6	28,4	105,8	121	14,4
Mato Grosso	400,3	279,4	-30,2	229,2	120,1	-47,6	23,8	21,2	-11	25,2	25,7	2,1	122,2	112,4	-8
Mato Grosso Sul	475,7	302,9	-36,3	288,1	139,9	-51,4	27,1	19,3	-29	23,2	18,9	-18,3	137,2	124,7	-9,1
Minas Gerais	510,4	292,9	-42,6	297,6	120,3	-59,6	34,3	18,7	-45,6	33	21,9	-33,6	145,5	132,1	-9,3
Paraná	562,7	311	-44,7	327,5	122,9	-62,5	42,5	22,4	-47,2	26,4	23	-12,9	166,4	142,8	-14,2
Paraíba	332,8	337,6	1,4	183,7	155	-15,6	20,1	16,5	-17,6	29,5	34,6	17,5	99,6	131,5	32
Pará	374,7	304,6	-18,7	216,4	135,3	-37,5	20	18,8	-5,8	21,9	32	46	116,5	118,5	1,7
Pernambuco	475,4	339,7	-28,5	286,5	159,9	-44,2	24,4	27,5	12,7	39,2	28,9	-26,2	125,3	123,4	-1,5
Piauí	331,1	275	-16,9	202,3	133,1	-34,2	18,7	11,6	-37,6	25	28,1	12,2	85,1	102,2	20,1
Rio Grande do Norte	286	295,7	3,4	150,4	130,2	-13,5	12,7	11,2	-11,4	30,6	29,2	-4,6	92,3	125,2	35,6
Rio Grande do Sul	565,4	321,7	-43,1	283,5	109	-61,6	49,6	27,1	-45,5	23,7	23,8	0,4	208,6	161,9	-22,4
Rio de Janeiro	680,4	367,2	-46	407,4	168,7	-58,6	33,9	19,2	-43,4	46,3	30,6	-33,9	192,7	148,7	-22,9
Rondônia	431	198,7	-53,9	249	84,2	-66,2	28	14,3	-48,9	28,8	18,3	-36,5	125,3	81,9	-34,6
Roraima	632,7	229,8	-63,7	348,5	97,3	-72,1	32,5	11,5	-64,8	54	26,8	-50,3	197,8	94,3	-52,3
Santa Catarina	514,9	291	-43,5	270,5	108,1	-60	47	21,2	-54,9	24,6	17,8	-27,4	172,8	143,9	-16,7
Sergipe	366,2	279	-23,8	189,3	119,9	-36,7	21,1	15,9	-24,7	40,6	30,6	-24,5	115,1	112,6	-2,2
São Paulo	556,9	327,7	-41,2	316,5	148,4	-53,1	31,1	19,2	-38,2	31,8	17,3	-45,6	177,6	142,8	-19,6
Tocantins	318,2	268,1	-15,8	193,2	128	-33,8	23,2	15	-35,6	22,1	28,5	29,1	79,7	96,6	21,2

PC: *Percentage of change*; DCNT: Doenças Crônicas não Transmissíveis.

### Fatores de risco

A Tabela 1 Suplementar mostra o SEV padronizado por idade para FR relacionados às DCNT no Brasil de 1990 a 2021. Entre esses anos, as maiores reduções de SEV para ambos os sexos combinados e todas as idades ocorreram para tabaco (-55,9%) e poluição do ar (-44,13%). Em contraste, aumentos no SEV foram observados para os seguintes FR: IMC elevado (+96,94%), alta glicemia de jejum (+53,28%) e baixa atividade física (+39,68%). Resultados semelhantes foram encontrados ao analisar a população de 30 a 69 anos.

### Projeções para 2030 e 2050

Ao analisar as projeções dos SEV até 2050, observou-se que colesterol LDL, pressão arterial, glicose de jejum e IMC elevados; baixa atividade física; e riscos referentes a dieta/alimentação, temperatura e função renal apresentam tendência de aumento, entre 30 e 69 anos e em todas as idades. O tabagismo, embora em queda no início da série, tendeu a estabilizar entre 30 e 69 anos. O uso excessivo de álcool e a exposição à poluição do ar mostraram tendência de redução (Figura 1).

**Figura 1.** Tendências e projeções dos fatores de risco, segundo o *Summary Exposure Value* *, para Doenças Crônicas não Transmissíveis de 1990 a 2021 e projeções até 2050.

*Foi considerado o *Summary Exposure Value* (SEV).

A Figura 2 apresenta as tendências e projeções das taxas de mortalidade pelos 4 principais grupos de DCNT até 2050. As mortes prematuras por DCV mantêm uma tendência de declínio contínuo e, a partir de 2024/2025, passam a apresentar taxas inferiores às das neoplasias (estas últimas atingirão as taxas mais elevadas, dentre todas as DCNT, nas próximas décadas). As taxas de mortalidade por doenças crônicas respiratórias e diabetes mostram quedas mais discretas. Quando consideradas todas as faixas etárias, ocorrerão declínios das DCV, que se mantêm em primeiro lugar, seguidas de câncer e doenças respiratórias crônicas. Ainda, observa-se crescimento das taxas de diabetes, que ocuparão o terceiro lugar.

**Figura 2.** Tendências das taxas de mortalidade pelos quatro principais grupos de Doenças Crônicas não Transmissíveis e projeções para 2050, 1990-2021.

Por sua vez, a Figura 3 apresenta as tendências da probabilidade de morte prematura por essas doenças, com projeções para 2030 e 2050. As metas globais de redução da mortalidade prematura por DCNT em 30% até 2030, tomando como base os dados de 2015 a 2021, poderão não ser alcançadas caso se mantenha o comportamento observado nesse período.

**Figura 3.** Taxas de mortalidade e probabilidade de morte prematura por Doenças Crônicas não Transmissíveis: tendências (1990-2021) e projeções (2030 e 2050), 1990-2021.

## DISCUSSÃO

O estudo apontou declínios significativos na mortalidade por DCNT entre 1990 e 2021, reflexo da melhoria das condições gerais de vida, da ampliação do acesso aos cuidados em saúde - como a expansão da atenção primária^24^, os programas de distribuição de renda^25^ ^,^ ^26^, o acesso a medicamentos e o aprimoramento da assistência de média e alta complexidade -, bem como da redução de fatores de risco, como a prevalência do tabagismo e o aumento da prática de atividade física^4^. Destaca-se, ainda, o papel fundamental do SUS, que contribuiu para a melhoria de diversos indicadores e resultados de saúde^27^.

No entanto, esse declínio não foi contínuo ao longo do tempo; em 2016 e 2021 observou-se aumento nas taxas de mortalidade por DCNT. Estes são atribuídos ao aprofundamento das desigualdades socioeconômicas, às políticas de austeridade fiscal e ao subfinanciamento do SUS^5^ ^,^ ^28^. O aumento observado em 2021 foi atribuído aos efeitos da pandemia de COVID-19, que resultaram tanto em piora nos estilos de vida da população - com aumento do sedentarismo e dos sentimentos de tristeza e solidão^2^ ^,^ ^11^, além de piora na alimentação - quanto no aumento da mortalidade, em especial por doenças cardiovasculares^29^. Esse cenário pode ser explicado, em parte, pela interrupção ou redução do acompanhamento clínico e monitoramento de pessoas com DCNT durante a pandemia, além dos possíveis efeitos da COVID-19 longa, que podem ter contribuído para o agravamento do quadro de saúde e para o aumento da mortalidade por DCNT no período pós-pandemia^30^ ^,^ ^31^.

As maiores reduções nas taxas de mortalidade por DCV podem ser explicadas por diversos fatores, entre eles a expansão da atenção primária à saúde; os programas de distribuição de renda; a redução da prevalência do tabagismo; e a melhoria na assistência, tanto pelo aumento do acesso aos serviços de atenção primária^13^ quanto pela ampliação do acesso a medicamentos para o controle da hipertensão arterial^32^.

O estudo evidenciou que o câncer, em poucos anos, se tornará a principal causa de morte prematura por DCNT no Brasil, sinalizando a continuidade do processo de transição epidemiológica no país. As neoplasias já representam a segunda principal causa de morte na maioria dos países^21^. No Uruguai e na Argentina, o câncer é a principal causa de óbito^33^. No Brasil, estudos apontam tendência de aumento na incidência de alguns tipos de câncer entre 1990 e 2015, como câncer de pulmão em mulheres (+20,7%) e câncer de cólon e reto em homens (+29,5%)^34^. Essa mudança reflete alterações nos padrões de exposição a fatores de risco, nos avanços terapêuticos e nos sistemas de rastreamento e diagnóstico precoce^35^.

O diabetes está associado ao aumento da mortalidade por doenças cardiovasculares, com efeitos sistêmicos que impactam diversos órgãos e funções do corpo^23^. Há evidências consistentes de que o crescimento da prevalência do diabetes está fortemente associado ao aumento da obesidade na população^23^.

A significativa redução nas taxas de mortalidade por doenças respiratórias crônicas pode ser atribuída, em grande parte, à queda na prevalência do tabagismo^4^ ^,^ ^36^. Outros fatores que podem ter contribuído para essa tendência incluem a ampliação do acesso aos serviços de saúde, especialmente com a expansão da Atenção Primária à Saúde (APS) no país^37^.

De acordo com a análise por unidades federativas, os padrões apresentam certa semelhança entre os estados. Quanto a diabetes e neoplasias, observa-se uma tendência de aumento ou estabilização na maioria dos estados. Destaca-se que os estados das regiões Norte e Nordeste tendem a apresentar menores reduções ou até aumento nas taxas, o que pode estar relacionado às piores condições de vida, mas também às possíveis limitações na qualidade da informação dos sistemas de registro de mortalidade, especialmente em anos anteriores^13^ ^,^ ^38^ ^,^ ^39^.

### Fatores de risco

Os resultados apresentados apontam uma redução significativa dos principais FR, especialmente os comportamentais - com destaque para o tabagismo. Evidências indicam que o controle dos fatores de risco e das DCNT é mais efetivo quando há implementação de medidas regulatórias por parte do Estado^2^ ^,^ ^4^ ^,^ ^40^. No caso do tabaco, diversas ações regulatórias foram adotadas, como a adesão à Convenção-Quadro para o Controle do Tabaco (CQCT), em 2006; e o Decreto Presidencial n^o^ 8.262, de 2014, que ampliou as advertências nos maços de cigarro e regulamentou ambientes livres de fumo e taxação sobre produtos derivados do tabaco^4^ ^,^ ^13^. Entretanto, a partir de 2016, o país vivenciou um enfraquecimento das políticas públicas de controle do tabagismo, com estagnação das políticas de preços e impostos, bem como uma crescente interferência da indústria do tabaco nos ambientes político e jurídico, evidenciando a influência dos determinantes comerciais na saúde^8^ e resultando em pior desempenho das políticas^36^ ^,^ ^41^ ^,^ ^42^.

Fatores de risco metabólicos projetam riscos elevados no futuro, representando uma ameaça à saúde da população nos próximos anos. Na última década, o Brasil implementou importantes políticas de fortalecimento da APS, como a criação do Programa Mais Médicos, ampliação do acesso gratuito a medicamentos para DCNT, ações de promoção da saúde, além de programas de transferência de renda^6^ ^,^ ^32^.

 A obesidade constitui um grave problema de saúde pública no Brasil, com tendência de aumento nas prevalências entre a população adulta. Estima-se que aproximadamente um terço dos adultos das capitais brasileiras seja obeso e cerca de 60% apresentem excesso de peso^11^. Esse crescimento acelerado reforça a necessidade de ampliar o acesso ao tratamento da obesidade; incentivar a prática regular de atividade física; promover o aleitamento materno exclusivo; e avançar em políticas fiscais, como o aumento da tributação de alimentos ultraprocessados. Tais medidas visam fortalecer o acesso a uma alimentação saudável durante todo o curso da vida, conforme as diretrizes do Guia Alimentar para a População Brasileira^43^.

A alimentação inadequada tem projeção de aumento do SEV até 2050. Evidências mostram aumento de alimentos ultraprocessados e redução no consumo de alimentos in natura, como Frutas, Legumes, Verduras (FLV) e feijão, em decorrência de mudanças nos padrões alimentares, da crise econômica e adoção de políticas de austeridade a partir de 2016^5^ ^,^ ^44^.

As “melhores evidências” indicadas pela OMS para prevenção e controle das DCNT incluem medidas regulatórias e de promoção à saúde, como taxação de bebidas, tabaco, alimentos ultraprocessados, além de proibição de marketing de produtos nocivos à saúde^45^. Portanto, dependem de investimento em políticas públicas e de decisões políticas para avançar nas pautas propostas, inclusive enfrentando interesses dos *lobbies* industriais e comerciais^45^.

### Projeções para 2030 e 2050

O estudo indica que as projeções das taxas de mortalidade por DCNT para 2030 e 2050 sinalizam a possibilidade de não cumprimento das metas de redução estabelecidas. Esse cenário reforça a necessidade de manter um monitoramento contínuo, a fim de identificar possíveis mudanças no padrão das doenças, dos seus determinantes e fatores de risco, e ajustar oportunamente as estratégias de enfrentamento ao longo dos próximos anos.

#### Quais os desafios globais e nacionais para alcançar as metas em 2050?

O investimento em ações de promoção da saúde e prevenção das DCNT pode resultar em avanços significativos, desde que haja decisão política e implementação efetiva de medidas de prevenção^10^. Um exemplo concreto é a redução global do tabagismo em cerca de 25%, na maioria dos países, desde a adoção da CQCT a partir de 2007^10^ ^,^ ^46^. Na China, os níveis de partículas finas em suspensão no ar (PM2,5) foram reduzidos em quase 50% na última década, demonstrando a eficácia de políticas ambientais rigorosas^47^.

Nesse contexto, as medidas aprovadas no Brasil, referentes à reforma tributária de taxação de tabaco e refrigerantes, representam uma oportunidade estratégica para fortalecer medidas fiscais sobre produtos nocivos à saúde, com potencial impacto não apenas sobre as projeções de mortalidade até 2050, mas também sobre a sustentabilidade da prevenção e do controle das DCNT no longo prazo.

### Limitações do estudo

Dentre os limites do estudo, as projeções para 2030 e 2050 foram baseadas nas tendências observadas em anos anteriores, assumindo a continuidade dos padrões passados como parâmetro para o futuro. No entanto, o futuro é incerto e pode incluir eventos imprevisíveis, como demonstrado pela pandemia de COVID-19. Fontes de dados incompletas, por exemplo, o SIM, têm avançado em cobertura e melhoria da qualidade dos registros, embora persistam subnotificação e elevada proporção de códigos *garbage* ^*19*^. Adicionalmente, os determinantes sociais não são incorporados diretamente nas análises causais^48^.

O estudo aponta que as metas globais podem não ser alcançadas, acendendo um alerta para a necessidade urgente de fortalecimento das políticas públicas voltadas à prevenção e ao controle das DCNT. Destaca-se a importância de intensificar ações intersetoriais e estratégias de promoção da saúde, diagnóstico precoce, acesso oportuno ao tratamento e acompanhamento contínuo - especialmente no âmbito da APS.

## Material suplementar

Tabela 1
